# Identifying the spatiotemporal patterns and natural and socioeconomic influencing factors of PM_2.5_ and O_3_ pollution in China

**DOI:** 10.1371/journal.pone.0317691

**Published:** 2025-02-13

**Authors:** Dongsheng Zhan, Zichen Wang, Hongyang Xiang, Yukang Xu, Kan Zhou

**Affiliations:** 1 School of Management, Zhejiang University of Technology, Hangzhou, China; 2 China Academy of Housing and Real Estate, Zhejiang University of Technology, Hangzhou, China; 3 School of Management, Shanghai University, Shanghai, China; 4 Institute of Geographic Sciences and Natural Resources Research, Chinese Academy of Sciences, Beijing, China; 5 College of Resources and Environment, University of Chinese Academy of Sciences, Beijing, China; Southwestern University of Finance and Economics, CHINA

## Abstract

To promote collaborative governance of PM_2.5_ and O_3_ pollution, understanding their spatiotemporal patterns and determining factors is crucial to control air pollution in China. Using the ground-monitored data encompassing PM_2.5_ and O_3_ concentrations in 2019 across 337 Chinese cities, this study explores the spatiotemporal patterns of PM_2.5_ and O_3_ concentrations, and then employed the Multi-scale Geographically Weighted Regression (MGWR) model to examine the socioeconomic and natural factors affecting PM_2.5_ or O_3_ concentrations. The results show that PM_2.5_ and O_3_ concentrations exhibit distinct monthly U-shaped and inverted U-shaped temporal fluctuation patterns across Chinese cities, respectively. Spatially, both pollutants manifest spatial clustering characteristic and a certain degree of bivariate spatial correlation. Elevated PM_2.5_ concentrations are predominantly concentrated on north and central China, as well as the Xinjiang Autonomous Region, whereas higher O_3_ concentrations are distributed widely across north, east, and northwest China. The MGWR model outperforms traditional OLS and global spatial regression models, evidenced by its enhanced goodness-of-fit metrics. Specifically, the R^2^ values for the PM_2.5_ and O_3_ MGWR models are notably high, at 0.842 and 0.861, respectively. Socioeconomic and natural factors are found to have multi-scale spatial effects on PM_2.5_ and O_3_ concentrations in China. On average, PM_2.5_ concentrations show positively correlations with population density, the proportion of the added value of secondary industry in GDP, wind speed, relative humidity, and atmospheric pressure, but negatively relationship with per capita GDP, road density, urban greening, air temperature, precipitation, and sunshine duration. In contrast, O_3_ concentrations are also positively associated with population density, the proportion of the added value of secondary industry in GDP, energy consumption, precipitation, wind speed, atmospheric pressure, and sunshine duration, but negatively correlated with per capita GDP, road density, and air temperature. Our findings offer valuable insights to inform the development of comprehensive air pollution management policies in in developing countries.

## 1. Introduction

Air pollution incidents have seriously impaired public health, manifesting in adverse impacts on respiratory systems [1], and aggravating cardiovascular diseases, appendicitis, intestinal diseases, kidney diseases, and a variety of other health conditions [2–5]. According to the 2019 Bulletin of the State of China’s Ecological Environment, ambient particulate matter (PM_2.5_) and ozone (O_3_) are recognized as the predominant primary air pollutants, which substantially degrade environmental quality, pose a threat to human health, and amplify the societal burden. During China’s 13th Five-Year Plan period (2015–2020), a noticeable increase in PM_2.5_ concentrations was observed during autumn and winter seasons, resulting in recurrent episodes of severe air pollution, while O_3_ concentrations exhibited a steady increase during the summer months. These trends have resulted in a nationwide decrease in the number of days that meet acceptable air pollution standards. With an emphasis on high-quality urban development, there has been an increasing focus on combating air pollution, leading to improvements in ambient air quality. However, the average PM_2.5_ concentrations in China still surpass the safety thresholds recommended by the World Health Organization (WHO), and O_3_ pollution has also become an increasingly pressing concern.

From a national strategic perspective, air pollution control has been established as a top priority by the Ministry of Ecology and Environment in China and is a key component of the China’s 14th Five-Year Plan. The Chinese government’s “Opinions on Further Advancing the Tough Battle Against Pollution” calls for decisive measures to reverse the rising trend of O_3_ concentrations by 2025, with a clear goal of managing both PM_2.5_ and O_3_ pollution collaboratively. Therefore, it is crucial to explore the spatiotemporal distribution of PM_2.5_ and O_3_ pollution in China, as well as the factors influencing these patterns. Such research can provide critical insights into mitigating PM_2.5_ and O_3_ air pollution, informing the development of integrated air pollution governance strategies for Chinese cities.

Over the past few decades, numerous studies have investigated the spatiotemporal characteristics of a spectrum of air pollutant concentrations, encompassing PM_2.5_, PM_10_, O_3_, CO, NO_x_, and SO_2_. However, the majority of these studies have focused on analyzing the spatiotemporal characteristics of PM_2.5_ [6–8] and O_3_ [9–11] individually, with a notable scarcity of research that addresses the concurrent spatiotemporal variation of both pollutants [12,13]. Furthermore, existing literature indicates that PM_2.5_ and O_3_ concentrations exhibit distinct and divergent seasonal fluctuations and regional disparities. Regarding seasonal variations, PM_2.5_ concentrations typically experience a significant decrease in summer and reach their zenith in winter [14], while O_3_ concentrations follow an inverse trend, typically reaching their lowest point in winter and peaking in spring or summer [15,16]. Regarding regional variations, elevated PM_2.5_ concentrations are commonly observed to the east of the Hu Line, especially in the Bohai Rim region [17] and the Yangtze River Delta region [18]. O_3_ concentrations echo these regional patterns, exhibiting higher levels in areas with dense populations and robust economic development, as well as in high-altitude zones [19]. Similar trends have been observed in Europe and North America, where PM_2.5_ concentrations are known to spike during the cold season [20] while O_3_ rise during the warmer seasons [21]. Both PM_2.5_ and O_3_ pollutants are notably prevalent in densely populated areas [22]. However, the existing research has primarily concentrated on the spatiotemporal variation characteristics of individual air pollutants, leaving a research gap in understanding PM_2.5_ and O_3_ co-pollution dynamics. Furthermore, there is a scarcity of studies examining the concurrent spatiotemporal variation of PM_2.5_ and O_3_ concentrations at a national scale.

Another branch of literature has endeavored to identify the determinants of air pollution at different spatial scales. Concerning air pollutant types, the majority of current research has investigated the driving factors affecting PM pollution or the air quality index (AQI). Previous studies have indicated that the increase in PM_2.5_ and O_3_ pollution can be attributed, on one hand, to socioeconomic factors, with population size emerging as the most influential factor [23,24]. In addition, economic development elements such as industrial composition, urbanization level, openness degree, and road infrastructure intensity have also significantly contributed to the spread of urban PM_2.5_ and O_3_ pollution [25–27]. On the other hand, climatic factors have also been shown to play a role in influencing PM_2.5_ and O_3_ concentrations, including wind speed, planetary boundary layer height (PBLH), temperature, water vapor mixing ratio, and solar radiation [13,28,29]. For instance, the North China Plain, known for its severe air pollution, exhibits heightened sensitivity to meteorological factors, particularly in the Taihang Mountains, where there are marked changes in terrain elevation [30]. Moreover, the upward trend of O_3_ in most Chinese cities is more strongly influenced by meteorological elements than PM_2.5_. Meteorological factors account for approximately 57–80% of the fluctuations in O_3_ concentrations, in contrast to only 20–33% of the changes in PM_2.5_ [31]. This is because PM_2.5_ is more significantly affected by the implementation of emission reduction measures for PM_2.5_ and its precursors [32].

To fill these research gaps, this study utilized ground-monitored air quality data collected in 2019 across 337 Chinese cities at the prefecture-level and above to identify the spatial characteristics of PM_2.5_ and O_3_ concentrations. In addition, the study employed bivariate global spatial autocorrelation and bivariate local spatial autocorrelation, global regression models such as the Ordinary Least Squared (OLS) model, Spatial Lag Model (SLM) and Spatial Error Model (SEM), and the Multi-scale Geographically Weighted Regression (MGWR) model, to investigate the spatial heterogeneous effects of influencing factors on PM_2.5_ and O_3_ concentrations in China. This study contributes to the literature by providing valuable insights into the spatial patterns of co-pollution involving air pollutants, with a particular emphasis on comparing the influencing factors of PM_2.5_ and those of O_3_. The study effectively addresses research gaps by examining the multi-scale spatial variations influenced by socioeconomic and natural factors, thereby deepening our understanding on the influencing mechanisms of spatial disparities in air pollution across Chinese cities. Furthermore, it establishes a framework for the advancement of studies on spatiotemporal patterns and factors affecting air pollution in urban China. The practical implications of this study are twofold. Firstly, our study can inform and improve environmental public policies in China, specifically in the context of collaborative air pollutants governance. This strongly aligns with the strategic objective outlined in China’s 14th Five-Year Plan to “strengthen the collaborative management of PM_2.5_ and O_3_”. Secondly, the study can also furnish evidence to bolster spatial planning initiatives aimed at fostering the coordinated governance of different air pollutants.

## 2. Materials and methods

### 2.1. Variables selection and data sources

Numerous studies have established a strong correlation between various socioeconomic factors and air pollutant concentration, highlighting their significant influence. These factors include renewable energy consumption use, per capita GDP, the level of urbanization, per capita CO_2_ emissions, the level of urbanization, the proportion of fossil fuel consumption, the proportion of secondary industries, industrial structure, and road intensity, which are all crucial socioeconomic factors affecting air pollutant concentrations [25,33]. Natural factors, such as relative humidity, air temperature, wind speed, and precipitation, are also identified as significant predictors of air pollution concentrations [31]. Consequently, PM_2.5_ and O_3_ pollution concentrations are subject to human social and economic activities and natural factors. In response to these findings, this paper focuses on the intricate association between these factors and pollutants. The socioeconomic factors surveyed comprised population density (Popden), per capita GDP (PGDP), the proportion of the added value of secondary industry in GDP (Prosec), energy consumption (Energy), road density (Roadden), urban greening (NDVI), and other explanatory variables. The natural factors examined were mainly explained by air temperature (Tem), precipitation (Pre), wind speed (Ws), relative humidity (Rh), air pressure (Ap), and sunshine duration (Sd).

The dataset for PM_2.5_ and O_3_ pollution concentrations used in this study was sourced from the Atmospheric Environment Data Monitoring Center of the Ministry of Ecology and Environment (https://air.cnemc.cn:18007/, accessed on March 3, 2020). The scope of our study encompasses 337 cities within mainland China. However, due to the data availability limitations, this study excluded Hong Kong, Macao, Taiwan, and certain counties directly governed provinces. Socioeconomic data, including population density, per capita GDP, and the proportion of the added value of secondary industry in GDP, was obtained from reliable sources such as the 2020 China City Statistical Yearbooks and 2019 socioeconomic statistical bulletins. Road density data was derived from the road network data on the OSM website (https://download.geofabrik.de/asia/china.html). Energy consumption was acquired through an aggregation of nighttime lights in each city, with the nighttime lights data retrieved from the Earth Observation Group (https://eogdata.mines.edu/products/vnl/). The normalized difference vegetation index data, which measures urban greening, was obtained from the geospatial data cloud (http://www.gscloud.cn/). Natural elements data and monthly meteorological element in 2019 such as air temperature, precipitation, wind speed, relative humidity, air pressure, and sunshine duration, was acquired from the China Meteorological Administration Data Sharing Center (https://data.cma.cn/). Table 1 displays the descriptive statistics for these key variables. To enhance model fitting, a logarithmic transformation was performed on the original data, including population density, per capita GDP, and energy consumption.

**Table 1:** Descriptive statistics of variables.

	Variable	Code	Mean	SD	Min	Max
**Socioeconomic factors**	**Population density (p·km** ^−^ **²)**	Popden	456.68	678.60	0.38	7202.27
	**Per capita GDP (yuan)**	PGDP	61754.08	35467.54	14256	203489
	**Proportion of the added value of secondary industry in GDP (%)**	Prosec	38.34	10.74	9.10	68.80
	**Energy consumption**	Energy	60612.23	62899.86	2598	460518
	**Road density (km·km** ^−^ **²)**	Roadden	0.84	0.86	0.04	6.76
	**Urban greening**	NDVI	0.72	0.17	0.08	0.89
**Natural factors**	**Temperature (°C)**	Tem	14.03	5.62	−1.82	25.70
	**Precipitation (mm)**	Pre	77.04	45.74	3.19	202.76
	**Wind speed (m·s**^−**1**^)	Ws	2.16	0.37	1.24	3.19
	**Relative humidity (%)**	Rh	67.42	11.91	34.66	83.71
	**Air pressure (kPa)**	Ap	944.46	86.02	619.69	1016.66
	**Sunshine duration (%)**	Sd	168.65	45.91	76.91	265.76

**Fig 1:**
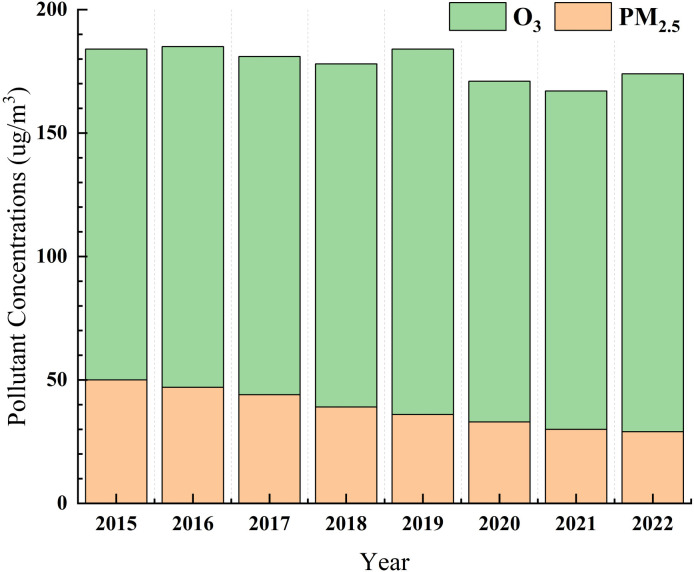
Annual average emission trends of PM_2.5_ and O_3_ concentrations in China.

The selection of PM_2.5_ and O_3_ pollution data from 2019 for this study is grounded in several compelling reasons. Firstly, the Chinese government has set ambitious targets for reducing PM_2.5_ and O_3_ emissions, aiming for a 10% reduction in PM_2.5_ concentrations in prefecture-level and above cities and effectively curbing the growth of O_3_ concentrations. However, Fig 1 shows that in 2019, the average emission of PM_2.5_ and O_3_ concentrations increased compared to 2018, prompting this study to investigate their causes. Secondly, the COVID-19 pandemic occurred in 2020 led to a three-year lockdown period in China and the unreliability of air pollution data compared to the normal years, potentially skewing the data’s scientific reliability. Lastly, the Ministry of Ecology and Environment in China has not publicly released an interface for downloading urban environmental quality data since 2022, thus preventing us from conducting research with more recent data. Considering the availability of data and the need for scientific precision, we have chosen year 2019 as the focal point for our subsequent analysis.

### 2.2. Research methods

We applied spatial statistical techniques to analyze the spatial association characteristics and distribution patterns of PM_2.5_ and O_3_. Our objective was to discover the spatial clustering patterns of PM_2.5_ and O_3_ by exploring both univariate and bivariate spatial correlations between them. Furthermore, global regression models, such as OLS, SEM, and SLM models, were employed to analyze the theoretical models. Additionally, MGWR model was introduced to examine the factors affecting the spatial variations of PM_2.5_ and O_3_ pollution concentrations across Chinese cities. Moreover, spatial heterogeneity in the relationship between the driving factors and pollution concentrations was examined.

#### 2.2.1. Spatial statistical methods.

The spatial statistical methods employed here involved bivariate global spatial autocorrelation and bivariate local spatial autocorrelation. The primary purpose of conducting the bivariate global spatial autocorrelation test is to evaluate spatial correlation within a spatial object for the same attribute variable across different periods or between different attribute variables. The Global Moran’s I Index ranges from −1 to 1. A value greater than 0 indicates a positive correlation in pollution levels between the two regions. The larger I is, the higher the similarity of the pollution in adjacent regions, leading to a greater likelihood of clustering due to similarity. When I is less than 0, the pollution between the two regions is negatively correlated. As the value of I decreases, the difference in pollution levels between adjacent regions increases, indicating a higher probability of pollution clustering due to these differences. When I is close to 0, regional pollution is randomly distributed, and no spatial correlation exists. The standardized statistic z-score can also be employed to assess correlations between regions. When z is greater than 0, the pollution between the two regions is positively correlated, and a higher value denotes a stronger positive correlation. Conversely, when z is less than 0, the pollution between the two regions is negatively linked. When z is 0, the pollution in the two areas is randomly distributed with no spatial correlation.

Bivariate local spatial autocorrelation discusses the spatial association patterns between attribute values of spatial units at different periods or among neighboring spatial units within a local area. The Local Moran’s I Index is not constrained within the [−1,1] range, with higher values indicating more substantial aggregation effects. When *I*_*i*_ exceeds 0, the adjacent area is characterized by aggregation in the same direction, either high or low. When *I*_*i*_ falls below 0, the characteristics of high-low or low-high aggregation patterns become evident.

#### 2.2.2. Global and local regression models.

Based on the extended STRIPAT model, this study examined associations of socioeconomic and natural factors on PM_2.5_ and O_3_ pollution concentrations, employing both global and local regression model. The global regression models applied in this study include OLS, SLM, and SEM, while the MGWR model is adopted as the local regression model. It should be noted that OLS regression ignores spatial interactions, spatial regression models overlook spatial non-stationarity [34]. While the GWR model can detect spatial non-stationarity, it neglects the differential scale effects of influencing factors, potentially leading to errors in regression results [35,36]. The MGWR model can effectively address the aforementioned issues and identify the scale differences of various influencing factors [37]. Therefore, we prefer to apply the MGWR model to explore the spatial scale effects of influencing factors.

(1) OLS model. The OLS model predicts dependent variables through a series of variables to quantify the relationship between socioeconomic and natural factors and PM_2.5_ or O_3_ concentrations. The formula was as follows:


Y = α + βX + ε

In this model, *Y* denotes the annual mean of the PM_2.5_ or O_3_ concentrations in each city; *α* and *β* are the constant terms and the estimated regression coefficients for the explanatory variables; *X* denotes the socioeconomic and natural factors that serve as explanatory variables; *ε* is the random error term.

(2) SLM. The spillover effect of surrounding areas on the research area was analyzed using the SLM, examining how the PM_2.5_ or O_3_ concentrations in the neighboring areas influence the research area. The formula was [38]:


Y = ρWY + βX + ε

Where *ρ* represents the regression coefficient of the spatial lag value of the dependent variable; *W* is the spatial weight matrix.

(3) SEM. The spatial dependence of certain factors in the error term, which may be ignored but still influence PM_2.5_ and O_3_ concentrations in the study area, was analyzed using the SEM. The formula was [38]:


Y = βX + ε, ε = λWε + μ

Where *λ* represents the regression coefficient of the spatial error, capturing the spatial dependence; *μ* denotes the random error term assumed to follow a normal distribution.

(4) MGWR model. Apart from the global regression model analysis, this paper also focused on analyzing the spatial heterogeneity of different influencing factors affecting PM_2.5_ and O_3_ pollution concentrations. This was achieved by applying a local regression model based on the MGWR model. By capturing the nuanced and varying relationship with variables across different spatial locations, the MGWR model improves upon the GWR model. The formula was as follows [35,36]:


$$Y_{i}\text{= }\alpha \text{(}u_{i}\text{,}v_{i}\text{) +}\overset{j\text{=1}}{\underset{k}{\sum }}\beta_{bwj}\text{(}u_{i}\text{,}v_{i}\text{)}X_{ij}\text{+ }\varepsilon_{i}$$

Where (*u*_*i*_, *v*_*i*_) represents the central coordinate at position *i*; *Y*_*i*_ denotes the attribute value at position *i*; *bwj* is the bandwidth used by the regression coefficient of the *j*^*th*^ variable; *β*_*bwj*_(*u*_*i*_, *v*_*i*_) is the regression coefficient of the *j*^*th*^ variable at the position *i*; *α*(*u*_*i*_, *v*_*i*_) and *ε*_*i*_ are the intercepts and error terms of the model at position *i*, respectively.

## 3. Results analysis

### 3.1. Descriptive statistical analysis

Table 2 presents the average annual PM_2.5_ concentrations in Chinese cities fr the year 2019, recorded as 36.83 ± 14.13 μg/m^3^. Notably, this value exceeds the high air quality threshold of 35 μg/m^3^, as outlined in the technical provisions of the AMBIENT AQI (2012). In this particular year, the range of PM_2.5_ concentrations spanned from a minimum of 6.55 ug/m^3^ in Ngari to a maximum of 108.68 ug/m^3^ in Hotan.

**Table 2:** Descriptive statistics of PM_2.5_ and O_3_ in China.

Variable	Mean	SD	Min	Max	N
**PM**_2.5_	36.83	14.13	6.55	108.68	337
**O**_3_	92.38	13.00	57.71	122.50	337

The average O_3_ concentrations in Chinese cities was 92.38 ± 13.00 μg/m^3^ (Table 2), which is close to the high air quality threshold of 100 ug/m^3^ stipulated in the technical provisions of the AMBIENT AQI (2012). Among the cities surveyed, Jixi City recorded the lowest O_3_ concentrations at 57.71 ug/m^3^, while Jincheng City had the highest O_3_ concentrations, reaching 122.50 ug/m^3^.

### 3.2. Temporal distribution patterns of PM_2.5_ and O_3_ pollution

**Fig 2:**
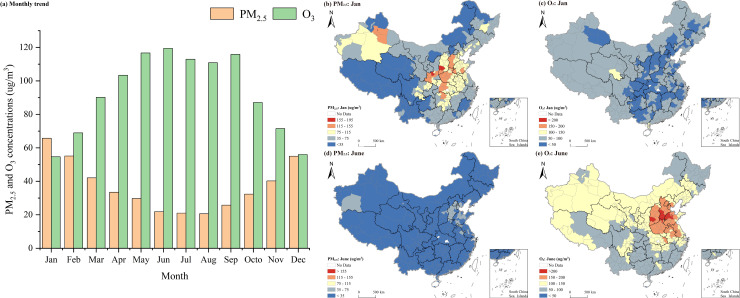
Temporal patterns of PM_2.5_ and O_3_ concentrations across Chinese cities. Note: Figures were based on the standard map from the Standard Map Service website of the Ministry of Natural Resources of the People’s Republic of China (http://bzdt.ch.mnr.gov.cn), with no modifications to the boundaries of the base map. The same applies below.

Fig 2 depicts the monthly temporal patterns of PM_2.5_ and O_3_ pollution concentrations across Chinese cities. Throughout the year, the temporal distribution of PM_2.5_ concentrations exhibited a U-shaped trend, commencing with a decrease and followed by an increase. In contrast, the temporal distribution of O_3_ pollution concentrations adhered to an inverted U-shaped pattern, initiating with an increase and then followed by a decrease. The examination of PM_2.5_ temporal distribution unveiled distinct features. Notably, PM_2.5_ pollution was significantly more severe during the winter months, with concentrations exceeding 50 ug/m^3^ in January, February, and December. Conversely, summer months saw relatively lower PM_2.5_ concentrations, ranging from 20 to 22 ug/m^3^ between June and August. During the transitional seasons of spring and autumn, the PM_2.5_ pollution concentrations remained within a moderate range.

The temporal distribution of O_3_ concentrations exhibited distinct patterns. From May to September, O_3_ concentrations in Chinese cities were at their highest, exceeding 110 ug/m^3^, peaking during and around the summer season. In contrast, winter months (January, February, and December) saw relatively lower O_3_ concentrations, fluctuating between 54 and 69 ug/m^3^. For the rest of the year, O_3_ concentrations fell within the intermediate range of 70–110 ug/m^3^. Moreover, a comparison of Fig 2b and 2e clearly shows that the regions most significantly affected by both pollutants are mainly located in north China, central plains, and south part of the Xinjiang Autonomous Region (Xinjiang).

### 3.3. Spatial distribution patterns of PM_2.5_ and O_3_ pollution

**Fig 3:**
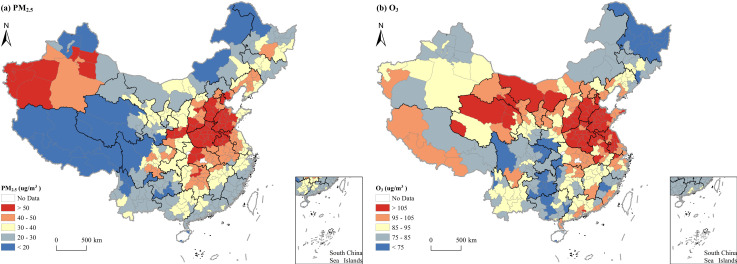
Spatial distribution of PM_2.5_ and O_3_ concentrations across Chinese cities.

Fig 3a illustrates the spatial distribution patterns of PM_2.5_ pollution concentrations in Chinese cities, which exhibits a higher concentration in the north and lower in the south, as well as a higher concentration in the east and lower in the west. Notably, areas with high PM_2.5_ pollution were concentrated in north and central China, and Xinjiang, with the Beijing-Tianjin-Hebei region being the most severely polluted. Regions where the PM_2.5_ pollution concentrations exceeding 50 ug/m^3^ were predominantly observed in south Hebei, north Shaanxi, north Anhui, southwest Shandong, central Hubei, and Henan. Additionally, PM_2.5_ pollution was also severe in northeast China, Xinjiang, Jiangsu, Hunan, Shaanxi, and Sichuan provinces, with the average annual concentrations ranging from 41 to 50 ug/m^3^. Conversely, areas with PM_2.5_ pollution concentrations below 30 ug/m^3^ were primarily located in south China, southwest China, the Inner Mongolia Autonomous Region (Inner Mongolia), parts of northeast China, and the Qinghai-Tibet plateau.

Fig 3b shows a north-south divide in O_3_ pollution concentrations in Chinese cities. High O_3_ pollution areas were mainly concentrated in north, east, and northwest China. Specifically, high pollution areas with annual average O_3_ concentrations exceeding 105 ug/m^3^ were mainly found in west Inner Mongolia, south Shanxi, southwest Hebei and Shandong, and parts of Henan and Qinghai, Jiangsu, and Anhui. High pollution areas with annual average O_3_ concentrations ranging from 95 to 105 ug/m^3^ were primarily situated in central and north China, as well as parts of Qinghai, Gansu, Inner Mongolia, and some coastal cities in the eastern regions. In contrast, low pollution areas with annual average O_3_ concentrations below 75 ug/m^3^ were predominantly located in south, southwest, and northeast.

### 3.4. Spatial clustering patterns of PM_2.5_ and O_3_ pollution

To further investigate spatial clustering patterns and correlations between PM_2.5_ and O_3_ concentrations, spatial statistical analysis of both PM_2.5_ and O_3_ concentrations in China was conducted using ArcGIS 10.8.

#### 3.4.1. Global spatial clustering patterns analysis.

Table 3 presents that the univariate Global Moran’s I value for PM_2.5_ and O_3_ concentrations were 0.6895 and 0.7732, respectively, indicating strong evidence of spatial clustering for each pollutant. Furthermore, the bivariate global Moran’s I value for the two pollutants is also significantly positive, with a result of 0.4388, suggesting a certain degree of spatial correlation between the two pollutants, forming a co-clustering pattern in space. Specifically, areas with high pollution concentrations were likely to be found near other areas with similarly high pollution concentrations.

**Table 3:** Global spatial associations of PM_2.5_ and O_3_ across Chinese cities.

Variable	Global Moran’s I	z-scores	p-values	Spatial adjacency weight
**PM**_2.5_	0.6895***	20.3917	0.0000	Queen, Geoda
**O**_3_	0.7732***	22.2041	0.0000	Queen, Geoda
**PM**_2.5_ **and O**_3_	0.4388***	28.9683	0.0000	Queen, Geoda

#### 3.4.2. Local spatial autocorrelation analysis.

Although the Global Moran’s I index offers a general understanding of the spatial correlation between PM_2.5_ and O_3_ pollution concentrations, it fails to fully capture the localized spatial clustering patterns or the heterogeneous distribution characteristics of these pollutants. Therefore, a local spatial autocorrelation analysis is necessary. Fig 3 reveals the specific spatial patterns of PM_2.5_ and O_3_ pollution concentrations.

**Fig 4:**
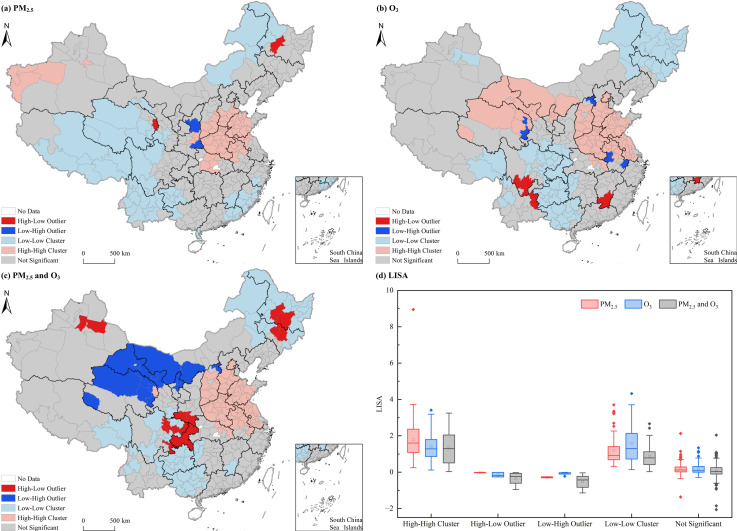
Spatial distribution patterns of LISA for PM_2.5_ and O_3_ across Chinese cities.

Fig 4a depicts the local spatial autocorrelation of PM_2.5_ pollution concentrations with distinct patterns. The results show that High-High (H-H) Clusters were predominantly concentrated in central and parts of north China, as well as west Xinjiang, with notable locations including Xi’an, Lianyungang, and Jingzhou. Conversely, Low-Low (L-L) Clusters were primarily observed at the junction of Heilongjiang and Inner Mongolia, southwest China, and the intersection of Fujian, Guangdong, and Jiangxi Provinces. Specific L-L Cluster regions included the Greater Hinggan Mountains, Hulunbuir, Heyuan, and Lishui. High-Low (H-L) outliers were observed in Suihua and Haidong City, while Low-High (L-H) Outliers were identified in Yanan and Shangluo City.

Fig 3b illustrates the local spatial autocorrelation of O_3_ pollution concentrations. H-H Custers were mainly observed in north and parts of central China, as well as at the junction of Gansu, Qinghai, and Inner Mongolia, with key regions including Beijing, Shijiazhuang, Jinan, and Haixi. L-L Clusters were primarily concentrated in northeast and southwest China, including Harbin, Changji, Chongqing, and Guilin. L-H Outliers were mainly located in Datong, Xining, Huangnan, Lu’an, and Xuancheng, while H-L Outliers were found in Ganzhou, Liangshan, and Qujing.

Fig 4c presents the bivariate local spatial autocorrelation of PM_2.5_ and O_3_ pollution concentrations. H-H Clusters, representing to regions with elevated levels of both pollutants, were predominantly concentrated in north and parts of east China, covering Shanxi, south Hebei, Beijing, Shandong, north Anhui, Jiangsu, and other provinces. Notable cities within these clusters include Shijiazhuang, Nanjing, Hefei, and other cities. Conversely, L-L Clusters, depicting regions with lower concentrations of both pollutants, were mainly observed in northeast and southwest China, with cities such as Ganzhou, Bijie, Dali, and Nyingchi. L-H Outliers, indicating the areas with low PM_2.5_ concentrations but high O_3_ concentrations, were primarily located in the border areas of Qinghai, Gansu, and Inner Mongolia, including cities such as Datong, Ordos, Wuwei, and Hainan Prefecture. H-L Outliers referring to regions with high PM_2.5_ concentrations but low O_3_ concentrations, were mainly concentrated in some parts of northeast China, the junction of southwest China and central China, and some areas of Xinjiang, including cities like Harbin, Changchun, Chongqing, and Hanzhong.

### 3.5. Influencing factors of spatial heterogeneity of PM_2.5_ and O_3_ pollution

This paper focused on the integrated association of socioeconomic and natural factors on the spatial heterogeneity of PM_2.5_ and O_3_ concentrations in urban areas across China. A comparative analysis was conducted using various global regression models, including the OLS model, SLM, and SEM, as well as a local regression model, the MGWR model. To assess the effectiveness of the model, their goodness of fit was evaluated using metrics such as R^2^, Akechi Information Criterion (AIC), and natural log-likelihood function values. A higher R^2^, smaller AIC values, and larger log-likelihood function values indicate better model fit.

#### 3.5.1. Global regression models.

Table 4 presents the results from the global regression models, revealing that models incorporating spatial dependence, specifically the SLM and SEM, outperformed the OLS model in terms of goodness of fit. The inclusion of spatial factors in these models led to significant increases in both the R^2^ value and the log-likelihood function. Meanwhile, the AIC values noticeably decreased, underscoring the importance of considering spatial factors. According to the diagnostic criteria proposed by Anselin for spatial regression model selection [38], the Lagrange Multipliers (LM) of the SLM and SEM were assessed and were both found to be significant, but the robust LM test of the SLM was deemed a more appropriate choice than the SEM (Table 5). Therefore, the subsequent analysis was based on the results of the SLM for PM_2.5_ and O_3_ pollution.

**Table 4:** Global regression models of PM_2.5_ and O_3_ concentrations in China.

Model	PM_2.5_			O_3_		
	OLS	SLM	SEM	OLS	SLM	SEM
Variable	Coef	Coef	Coef	Coef	Coef	Coef
**Constant**	75.060***	42.872***	−22.982	89.871***	21.884*	71.136***
**Popden**	5.370***	3.740***	4.129***	4.250***	1.335***	0.691
**PGDP**	−3.243**	−2.522**	−2.835**	1.162	−0.472	−0.706
**Prosec**	−0.024	−0.010	0.010	0.104***	0.070***	0.056**
**Energy**	−0.209	0.546	1.450**	−1.428*	0.133	0.611
**Roadden**	−1.895**	−1.651**	−2.060**	1.689*	0.140	−0.583
**NDVI**	−22.831***	−13.453***	−4.647	−11.138**	−5.703*	−11.606*
**Tem**	−0.156	−0.156	−0.179	0.835***	0.191	0.338
**Pre**	−0.126***	−0.041**	−0.027	−0.098***	−0.016	0.001
**Ws**	−7.293***	−5.064***	−5.565**	−1.061	−0.291	−1.420
**Rh**	−0.554***	−0.260***	−0.040	−0.226*	−0.044	0.009
**Ap**	0.079***	0.027***	0.084***	−0.014	−0.005	0.000
**Sd**	−0.117***	−0.041**	−0.019	0.064**	0.027	0.134***
***ρ*/*λ***		0.607***	0.837***		0.764***	0.852***
**R** ^2^	0.585	0.738	0.770	0.461	0.778	0.785
**AIC**	2469.55	2346.34	2339.16	2501.67	2257.7	2264.43
**Log-likelihood**	−1221.77	−1159.17	−1156.582	−1237.84	−1114.85	−1119.217

Note: *, **, *** indicate p < 0.1, p < 0.05, p < 0.01.

**Table 5:** Model test of SLM and SEM.

Test	PM_2.5_		O_3_	
	Value	Prob	Value	Prob
**LM(SLM)**	133.240	0.000	266.720	0.000
**RLM(SLM)**	34.959	0.000	94.040	0.000
**LM(SEM)**	99.234	0.000	176.889	0.000
**RLM(SEM)**	0.953	0.329	4.209	0.040

The results from the SLM of PM_2.5_ concentrations reveal a significantly positive spatial lag coefficient *ρ* value of 0.607, indicating a substantial spatial spillover effect of PM_2.5_ pollution across urban areas. Several factors, including population density, per capita GDP, road density, urban greening, precipitation, wind speed, relative humidity, air pressure, and sunshine duration, were found to significantly influence PM_2.5_ concentrations. Among these, population density exerted a notable positive influence on PM_2.5_ concentrations greatly, while the other explanatory variables had a significant negative effect.

Regarding the effect strength on PM_2.5_ pollution, the regression coefficient for population density was 4.129, meaning that a 1% increase in the logarithm of population density in a region corresponds to a 4.129% increase in PM_2.5_ concentrations. This suggests that the intensification of human social and economic activities in densely populated areas contributes to higher emissions of air pollutants, worsening local PM_2.5_ concentrations. Socioeconomic variables, such as per capita GDP, road density, and urban greening, exhibited significant negative effects on PM_2.5_ pollution, with corresponding regression coefficients of −2.522, −1.651, and −13.453, respectively. This implies that for every 1% increase in these variables in a region, the PM_2.5_ concentrations could decrease by 2.522%, 1.651%, and 13.453%, respectively. These results highlight the important roles of economic development level, road density, and urban greening in reducing PM_2.5_ pollution. Additionally, natural factors like precipitation, wind speed, relative humidity, air pressure, and sunshine duration demonstrated significant negative associations with PM_2.5_ pollution. The corresponding regression coefficients for these variables were −0.041, −5.064, −0.260, −0.027, and −0.041. This means that for every 1% increase in these natural factors in a region, the corresponding PM_2.5_ concentrations could decrease by 0.041%, 5.064%, 0.260%, 0.027%, and 0.041%, respectively. This suggests that favorable natural factors such as precipitation, wind speed, relative humidity, air pressure, and sunshine duration can help mitigate the local emissions.

The spatial lag coefficient *ρ* value for O_3_ concentrations in the SLM was determined to be 0.764, which is significantly positive. This suggests a stronger spillover effect of O_3_ pollution compared to PM_2.5_. The analysis of each explanatory variable revealed that socioeconomic factors, including population density, proportion of the added value of secondary industry in GDP, and urban greening, exhibited significant associations with O_3_ pollution in Chinese cities. The corresponding regression coefficients were 1.335, 0.070, and −5.703. This means that a 1% increase in population density and the proportion of the added value of secondary industry in GDP would be associated with a 1.335% and 0.070% increase in O_3_ pollution concentrations, respectively. In contrast, a 1% increase in urban greening would lead to a 5.703% decrease in O_3_ pollution concentrations. These findings suggest that higher population density and a greater proportion of the added value of secondary industry in GDP are linked to higher O_3_ pollution concentrations, while increased urban greening is associated with a reduction in local O_3_ pollution concentrations.

#### 3.5.2. Local regression model.

We employed the MGWR model to explore the variability of factors affecting PM_2.5_ and O_3_ concentrations across different regions. Tables 6 and 7 demonstrate that the MGWR model outperformed other regression models, including OLS, SLM, and SEM, in terms of goodness of fit. This indicates that the MGWR model offers superior explanatory power for analyzing these associations. Specifically, its goodness of fit R^2^ and natural log-likelihood function values were significantly higher than those of the global regression models, while its AIC values were significantly lower. These results suggest that local regression model is better suited for capturing the relationships between variables. Taking into account the spatial heterogeneity of socioeconomic and natural factors is crucial for gaining a comprehensive understanding of their interaction.

**Table 6:** Parameter estimates and bandwidth of MGWR model for PM_2.5_ concentrations.

Variable	Mean	STD	Min	Median	Max	Bandwidth
**Constant**	0.302	0.016	0.274	0.299	0.355	329
**Popden**	0.440	0.203	−0.009	0.389	1.090	63
**PGDP**	−0.041	0.148	−0.436	−0.056	0.307	60
**Prosec**	0.084	0.049	−0.082	0.103	0.121	327
**Energy**	0.021	0.005	0.014	0.020	0.044	335
**Roadden**	−0.102	0.006	−0.109	−0.103	−0.075	335
**NDVI**	−0.031	0.036	−0.144	−0.024	0.016	278
**Tem**	−0.528	0.317	−0.925	−0.555	0.083	165
**Pre**	−0.291	0.204	−0.805	−0.243	0.057	73
**Ws**	0.042	0.245	−0.750	0.056	0.589	56
**Rh**	0.036	0.164	−0.254	0.000	0.314	94
**Ap**	0.635	0.009	0.614	0.635	0.649	335
**Sd**	−0.213	0.013	−0.238	−0.212	−0.176	329
**R** ^2^	0.842					
**AIC**	458.629					
**Log-likelihood**	−167.088					

**Table 7:** Parameter estimates and bandwidth of MGWR model for O_3_ concentrations.

Variable	Mean	STD	Min	Median	Max	Bandwidth
**Constant**	0.637	0.121	0.253	0.625	0.842	96
**Popden**	0.232	0.300	−0.338	0.294	0.852	44
**PGDP**	−0.068	0.005	−0.090	−0.067	−0.063	335
**Prosec**	0.173	0.092	−0.024	0.166	0.467	81
**Energy**	0.064	0.035	0.002	0.063	0.123	280
**Roadden**	−0.103	0.003	−0.106	−0.104	−0.089	335
**NDVI**	−0.014	0.047	−0.088	−0.011	0.098	292
**Tem**	−0.209	0.686	−0.971	−0.445	1.427	44
**Pre**	0.019	0.132	−0.125	−0.009	0.386	156
**Ws**	0.115	0.333	−0.349	0.027	0.816	56
**Rh**	0.091	0.009	0.075	0.089	0.116	335
**Ap**	0.454	0.003	0.443	0.454	0.458	335
**Sd**	0.455	0.008	0.425	0.457	0.466	335
**R** ^2^	0.861					
**AIC**	426.559					
**Log-likelihood**	−145.757					

**Fig 5:**
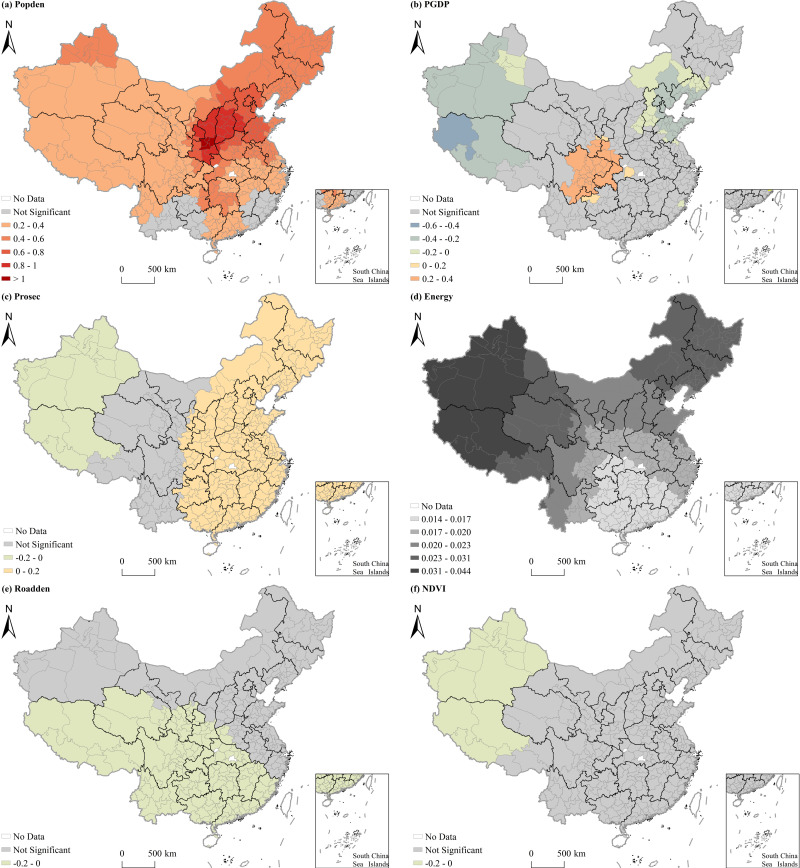
Spatial heterogeneous effects of socioeconomic factors on PM_2.5_ concentrations in China. Notes: Warm hues denote areas with influencing factors significantly above 0, whereas cool shades signify those significantly below 0. Gray is used for areas lacking significance, and white for areas devoid of data. This color scheme is consistent across subsequent figures.

**Fig 6:**
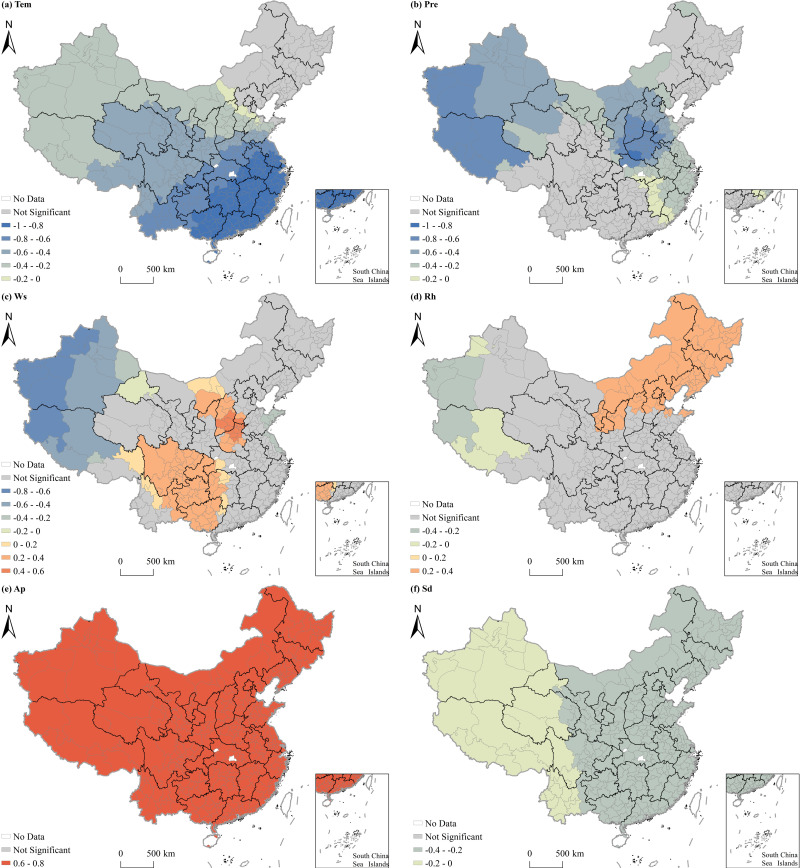
Spatial heterogeneous effects of natural factors on PM_2.5_ concentrations in China.

Table 6 shows the results of the MGWR model, highlighting the mean impact intensity of different factors on PM_2.5_ concentrations across Chinese cities. The findings indicate a positive correlation between PM_2.5_ concentrations and factors such as population density, the proportion of the added value of secondary industry in GDP, wind speed, relative humidity, and air pressure. However, PM_2.5_ concentrations were negatively correlated with per capita GDP, road density, urban greening, air temperature, precipitation, and sunshine duration. Figs 5 and 6 provide the geographical maps illustrating the associations between socioeconomic and natural factors on PM_2.5_ concentrations, respectively. Areas shaded in gray in these maps indicate regions where the impact of the explanatory variable is not significant. The results reveal that, with the exception of energy consumption, all other influencing factors exhibited a substantial effect on PM_2.5_ concentrations, with the impact intensity varying across regions. Explanatory variables such as the proportion of the added value of secondary industry in GDP, road density, air pressure, and sunshine duration recorded a large bandwidth exceeding 300, indicating global associations with PM_2.5_ pollution. The bandwidth of explanatory variables such as urban greening and air temperature ranged from 165 to 278, suggesting regional impacts. Meanwhile, variables like population density, per capita GDP, Pre, wind speed, and relative humidity have a bandwidth ranging from 56 to 94, signifying more localized influences.

The impact of Population density on PM_2.5_ concentrations was found to be positive, with a higher intensity of influence observed in the east regions compared to the west. Notably, Shaanxi and Shanxi Provinces experienced the highest impact intensity. Per capita GDP significantly affected PM_2.5_ concentrations in regions such as the Bohai Rim, Sichuan-Chongqing, Tibet, and Xinjiang. Interestingly, a positive correlation between per capita GDP and PM_2.5_ pollution was observed in the Sichuan-Chongqing region, while other areas showed negative effects. The impact of the proportion of the added value of secondary industry in GDP on PM_2.5_ pollution was mixed. Specifically, there was a positive correlation in the east regions, with impact intensity increasing from west to east. In west China, the influence was negative, with impact intensity decreasing from west to east. In addition, road density and urban greening both showed significant negative effects on PM_2.5_ concentrations. Road density’s impact was primarily observed in southwest China, with the impact intensity gradually decreasing from southwest to east. Urban greening had a more significant impact in northwest Xinjiang and Tibet, with the impact intensity falling from west to east.

Air temperature negatively affected PM_2.5_ concentrations, with impact intensity decreasing from the north to the south across China. Thus, rising air temperature in the northern region of the country may greatly promote PM_2.5_ pollution control. Both precipitation and wind speed also had negative effects on PM_2.5_ concentrations. Precipitation exhibited the greatest influence in Xinjiang, Tibet, and the surrounding areas of Henan Province, while wind speed showed a more substantial impact on Xinjiang and Tibet due to the diffusion and dilution of PM_2.5_, leading to a reduction of regional pollution. In contrast, wind speed had the opposite influence on PM_2.5_ pollution in southwest and central China, where it was exhibited positively. This is primarily due to high local levels of PM_2.5_, which disperse to nearby areas under strong wind conditions. In north and northeast China, Ningxia, and Shaanxi, relative humidity significantly showed a positive relationship with PM_2.5_ pollution. Conversely, Xinjiang and west Tibet demonstrated an inverse relationship between relative humidity and PM_2.5_ pollution. Air pressure positively influenced PM_2.5_ pollution, with intensity decreasing from west to east. Finally, sunshine duration had a notable inverse relationship with PM_2.5_ pollution in Xinjiang, Tibet, and Qinghai, with pollution levels decreasing as sunshine duration increased. However, this effect was less pronounced in regions such as Aksu, Hetian, Kashgar, and Kizilsuzhou in Xinjiang.

**Fig 7:**
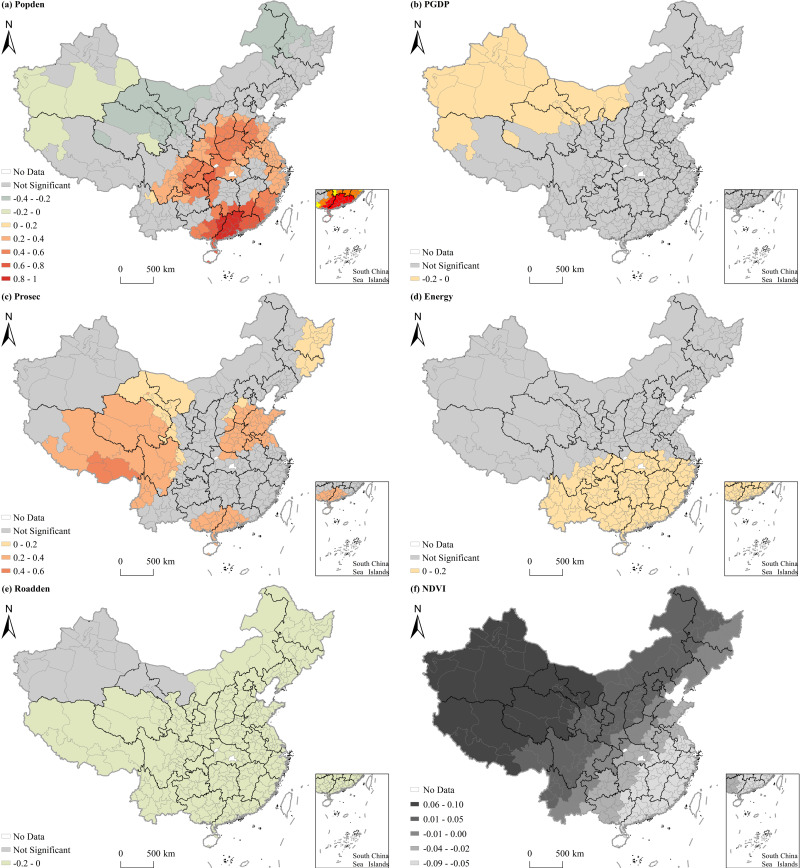
Spatial heterogeneous effects of socioeconomic factors on O_3_ concentrations in Chinese cities.

**Fig 8:**
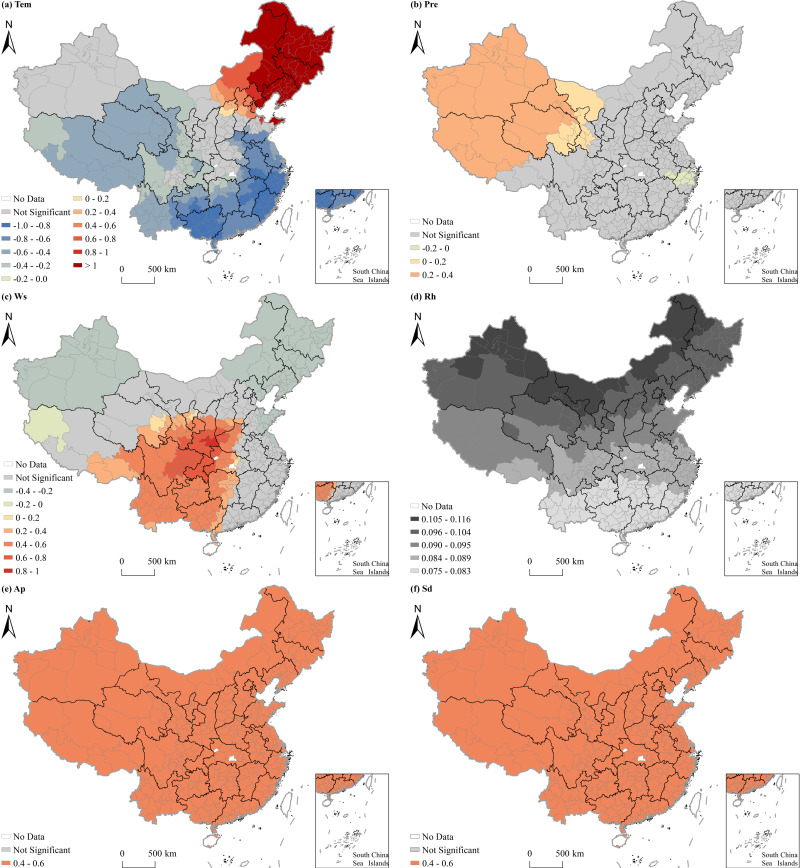
Spatial heterogeneous effects of natural factors on O_3_ concentrations in Chinese cities.

Table 7 presents the findings of the MGWR model on the variations in O_3_ concentration among Chinese cities. Regarding the average impact effect, O_3_ pollution concentrations were positively correlated with explanatory variables such as population density, the proportion of the added value of secondary industry in GDP, energy consumption, precipitation, wind speed, air pressure, and sunshine duration, and negatively correlated with explanatory variables such as per capita GDP, road density, and air temperature. Figs 7 and 8 demonstrate the diverse spatial impacts of socioeconomic and natural factors on O_3_ concentrations, respectively. Based on the visualization outcomes, it was observed that, aside from urban greening and relative humidity, all other influencing factors showed influence on O_3_ distribution significantly, with regional variations in the intensity of their impact. Explanatory variables such as per capita GDP, road density, air pressure, and sunshine duration had large bandwidths exceeding 300, indicating a global influence on the distribution of O_3_ pollution. Energy consumption and precipitation, with bandwidths of 156 and 292, demonstrated regional power. Population density, the proportion of the added value of secondary industry in GDP, air temperature, and wind speed had bandwidths varying from 44 to 81, signifying localized effects on the distribution of O_3_ pollution.

The influence of population density on O_3_ pollution varied significantly across regions. It had a positive impact in south and central China, as well as Chongqing, but a negative influence in other areas. Per capita GDP exhibited a notable adverse effect on O_3_ pollution in northwest China, with the most intense impact observed in north Xinjiang. The proportion of the added value of secondary industry in GDP positively affected O_3_ concentrations, with the highest impact intensity in Henan and Shandong, south Guangxi and Guangdong, and the junction of several provinces or autonomous regions such as Tibet, Qinghai, and Sichuan. In south China, there was a significant positive correlation between energy consumption and O_3_ pollution, with impact intensity increasing from north to south. Moreover, road density had a noteworthy negative association with O_3_ pollution in the southern regions, with the effect intensifying gradually from north to south.

In north and northeast China, air temperature demonstrated a positive correlation with O_3_ pollution. However, in other regions, air temperature had a negative effect, with the impact intensity gradually increasing from the northwest to the southeast. Precipitation had a significant positive impact on O_3_ pollution in northwest China, particularly in Xinjiang. Wind speed positively influenced O_3_ pollution in southwest and central China, with the highest intensity in Sichuan, Chongqing, and Shaanxi. Conversely, wind speed negatively affected O_3_ pollution in Xinjiang and northeast China. Air pressure had a positive influence on O_3_ pollution, with intensity decreasing and then increasing from west to east. Lastly, sunshine duration positively impacted O_3_ pollution, with intensity gradually rising from north to south.

## 4. Discussion

Consistent with previous studies [15,17,39–41], our study also observed notable seasonal variations in urban air pollution across China. Specifically, PM_2.5_ concentrations exhibited a U-shaped seasonal pattern throughout the year, with higher values observed during winter and lower values in summer. For most Chinese cities, this fluctuation can be largely attributed to hot and rainy summers that facilitate the dilution and reduction of particulate pollutants concentrations. Moreover, the reduced precipitation during winter leads to the formation of inversion layers that trap pollutants, along with the heightened increased energy consumption for heating in north China and other adverse factors, significantly contribute to the increase in PM_2.5_ pollution concentrations [42–46]. In contrast, O_3_ pollution displayed an inverted U-shaped seasonal variation over the year, with higher concentrations observed in summer and lower ones in winter. This was primarily attributed to the favorable conditions in summer conditions, such as higher temperatures, abundant sunshine, and dry air, which enhance the photochemical reactions between VOCs and NOx in the air, thereby easily exacerbating O_3_ pollution [47–50].

We also found that the two pollutants exhibited distinctive patterns of spatial clustering in cities. Specifically, north China and south Xinjiang were found to have a high concentration of PM_2.5_ pollution, while high O_3_ pollution was mainly found in north China, Qinghai, Inner Mongolia, and west Gansu. These observations are in line with the spatial patterns of air pollution in Chinese cities as reported by the majority of scholars [17,51], indicating that these regions had emerged as hotspots for hazardous air pollution in recent years. PM_2.5_ pollution distribution was primarily correlated with factors such as the presence of heavy industries, energy consumption, adverse climate conditions, and other comprehensive factors at the local level [52,53]. Alternatively, O_3_ pollution exhibited higher severity in northern cities, potentially attributed to the extended sunshine duration in summer within these regions, along with other contributing factors [11,30]. Additionally, a spatial correlation was observed between PM_2.5_ and O_3_ pollution, with regions of significant synergistic pollution primarily centralized in north China and parts of east China, underscoring the necessity for targeted control measures in these regions.

The superior fitting performance of the MGWR model, in comparison to the traditional OLS model and spatial regression models for both pollutants, provides evidence for the multi-scale spatial heterogeneity effects resulting from the interplay of socioeconomic and natural factors. This finding aligns with the existing literature [27,54,55], yet it also unveils new insights. Specifically, our study discerned no significant relationship between energy consumption and PM_2.5_ pollution, and neither urban greening nor relative humidity significantly affected O_3_ pollution. In terms of the impact indirection, PM_2.5_ pollution concentrations were positively associated with population density, the proportion of the added value of secondary industry in GDP, wind speed, relative humidity, and air pressure. Conversely, PM_2.5_ pollution concentrations were negatively associated with per capita GDP, road density, urban greening, air temperature, precipitation, and sunshine duration. These patterns can be ascribed to the strong correlation between PM_2.5_ pollution concentrations and human activity intensity, which leads to higher pollution concentrations in densely populated areas and those with a more substantial industrial base. Moreover, the increase in wind speed, relative humidity, and air pressure might contribute to the dispersion of neighboring air pollutants into the local areas or facilitate the formation of air pollution particles [56,57]. The increase in the level of economic development enhanced industrial structure, while an increase in road density improved commuting efficiency, which might reduce PM_2.5_ pollution. Air temperature, precipitation, and sunshine duration may help reduce the concentrations of air pollutants by increasing thermal convection and precipitation dilution [58–60].

Alternatively, O_3_ pollution concentrations showed a predominantly positive correlation with factors such as population density, the proportion of the added value of secondary industry in GDP, energy consumption, precipitation, wind speed, air pressure, and sunshine duration. In contrast, O_3_ pollution concentrations showed a negative correlation with per capita GDP, road density, and air temperature. Increasing precipitation and wind speed may reduce cloud cover, enhance solar radiation intensity, prolong sunshine duration, and strengthen photochemical reactions, leading to elevated O_3_ production and concentrations. The advancement of economic development supports the refinement of industrial structure, thereby contributing to the reduction of O_3_ concentrations [27,61]. Therefore, O_3_ concentration control could benefit from applying successful strategies used for PM_2.5_ strategies, such as reducing population density, boosting economic development, and limiting greenhouse gas emissions. On the other hand, for factors with different mechanisms or opposing influence, response policies must be tailored to their specific emission characteristics. Such targeted measures will help effectively curb emissions and mitigate further pollution. To successfully manage both PM_2.5_ and O_3_ concentrations, it is crucial to develop customized strategies that consider the distinct emission characteristics associated with each factor. This approach will ensure more effective air pollutant control and the implementation of sustainable solutions to reduce pollutant levels.

**Fig 9:**
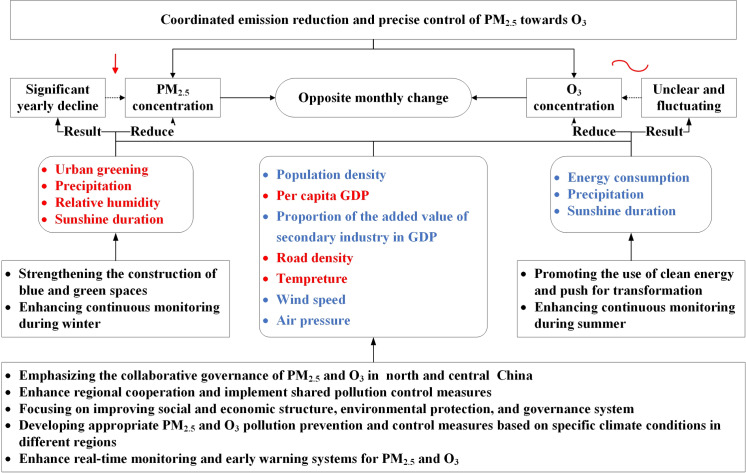
Policy roadmap of coordinated emission reduction and precise control of PM_2.5_ and O_3_. Notes: The red fonts in the picture indicate that the factor is negatively correlated with pollutant emissions, and therefore, its development needs to be promoted. On the other hand, the blue fonts show a positive correlation, and interventions are required to reduce pollutant emissions.

Our findings could provide several policy implications and outline a policy roadmap for coordinated emission reduction and precise control of PM_2.5_ and O_3_. This roadmap has the potential to guide the implementation of effective strategies aimed at combating air pollution and achieving substantial enhancements in air quality (Fig 9). Firstly, in order to effectively regulate and mitigate PM_2.5_ and O_3_ pollutants in high-pollution areas of north and central China, a suite of strategic actions is warranted. These include augmenting urban greening and vegetation efforts to absorb pollutants and ameliorate air quality. It is imperative to foster economic development and escalate GDP per capita, thereby facilitating a transition from heavy industry to less polluting sectors. Additionally, decreasing the secondary industry’s share in GDP can curtail industrial emissions, while the deployment of cloud-seeding techniques could serve to augment precipitation and reduce sunshine hours, consequently mitigating pollution levels. Secondly, to confront the pronounced spatial autocorrelation observed in PM_2.5_ and O_3_ pollution, there is a critical need to bolster regional cooperation and implement joint pollution control measures. This could entail the establishment of a cross-regional air quality monitoring and data sharing platform, the formulation of unified regional pollution control plans and objectives, the dissemination of pollution control technologies and experiences, and the enforcement of regional ecological compensation mechanisms. Thirdly, a focal point for regions should be the enhancement of their socioeconomic structures, environmental protection measures, and governance systems to address the common factors influencing both PM_2.5_ and O_3_. This is essential for collaborative pollution reduction efforts. Policies aimed at diminishing population and road density in highly polluted areas could reduce emissions and ameliorate air circulation. Furthermore, advocating for the use of clean energy and promoting the transformation of energy structures to reduce emissions and improve air quality. Lastly, it is of paramount to formulate tailored prevention and mitigation strategies for these pollutants, taking into account the unique and specific climate conditions of different regions. The impact of PM_2.5_ and O_3_ concentrations varies across areas due to disparate climate conditions. Regions that are particularly susceptible to natural climate factors should intensify their real-time monitoring and early warning systems for PM_2.5_ and O_3_ pollution, aiming to mitigate the seasonal fluctuations in pollution levels.

Nonetheless, our study also has some limitations Firstly, our study solely relied on cross-sectional data from 2019, failing to undertake a dynamic analysis of the spatiotemporal trends and underlying factors of air pollution over multiple timeframes. To address this shortcoming, future research should incorporate panel data analysis to enable a comparative assessment of spatiotemporal distribution patterns of PM_2.5_ and O_3_ pollution in Chinese cities across different historical periods. Secondly, our study had limitations related to data availability. The examination of influencing factors was constrained, and essential variables such as FDI investment, motor vehicle ownership, local environmental regulation intensity, and other related air pollutants or precursors were not fully considered. Future studies should incorporate additional relevant influencing factors of contaminants. Finally, our study’s temporal parameters were not sufficiently detailed. Since PM_2.5_ and O_3_ pollution displayed noticeable temporal variations, future investigations could conduct more refined studies on influence mechanisms based on meteorological factors across different periods.

## 5. Conclusion

Drawing on the ground-monitored air quality data from 337 Chinese cities at the prefecture-level and above in 2019, our study employed spatial autocorrelation analysis and the MGWR model to investigate the spatiotemporal distribution patterns of PM_2.5_ and O_3_ pollution. Furthermore, it quantified the spatial heterogeneity effects of both socioeconomic and natural factors on the concentrations of these pollutants. This study drew the following main conclusions.

(1) Chinese cities exhibited significant seasonal variations in both PM_2.5_ and O_3_ concentrations. The temporal trends revealed that PM_2.5_ concentrations exhibited a U-shaped pattern, while O_3_ concentrations followed an inverted U-shaped pattern throughout the year. Specifically, PM_2.5_ pollution levels were elevated in winter and reduced in summer, whereas O_3_ pollution was more pronounced during the summer season and less severe in winter.
(2) Both PM_2.5_ and O_3_ concentrations displayed distinct spatial clustering patterns across Chinese cities. Geographically, high-value PM_2.5_ concentrations were primally centered in north and central China, and several regions located in south Xinjiang. While regions with high-value O_3_ concentrations were mainly concentrated in north, east, and northwest China. PM_2.5_ and O_3_ concentrations demonstrated a certain degree of spatial correlation in China, with bivariate high-value regions for both pollutants primarily concentrated in north China.
(3) The MGWR model demonstrated superior goodness of fit compared to the OLS, SLM, and SEM models, yielding R^2^ values of 0.842 for the PM_2.5_ model and 0.861 for the O_3_ model. The parameter estimation results of the MGWR model revealed that there were spatially varying effects of both socioeconomic and natural factors on PM_2.5_ and O_3_ concentrations in Chinese cities at different scales. On average, PM_2.5_ concentrations exhibited positive correlations with population density, the proportion of the added value of secondary industry in GDP, wind speed, relative humidity, and air pressure, but negatively correlated with per capita GDP, road density, urban greening, air temperature, precipitation, and sunshine duration. Similarly, O_3_ concentrations were positively correlated with population density, the proportion of the added value of secondary industry in GDP, energy consumption, precipitation, wind speed, air pressure, and sunshine duration, but showed negative correlations with per capita GDP, road density, and air temperature.

## Supporting information

S1 File Data for Fig 5. (XLSX)

S2 File Data for
Fig 6.
(XLSX)

S3 File Data for Fig 7. (XLSX)

S4 File Data for Fig 8. (XLSX)
